# Home-based exercise rehabilitation experience for older adult patients with Parkinson’s disease and caregivers: a qualitative research

**DOI:** 10.3389/fpubh.2025.1735475

**Published:** 2025-12-22

**Authors:** Honglian Dong, Peishan Li, Li Xue, Xiao'e Jiang, Mohedesi Siyiti

**Affiliations:** 1 School of Nursing, Xinjiang Medical University, Urumqi, China; 2 Health Care Research Center for Xinjiang Regional Population, Urumqi, China; 3 Department of Neurology III, The Second Affiliated Hospital of Xinjiang Medical University, Urumqi, China; 4 Xinjiang Key Laboratory of Neurological Diseases Research, Urumqi, China

**Keywords:** Parkinson’s disease, older adults, caregivers, home-based exercise rehabilitation, experience, qualitative research

## Abstract

**Objective:**

To understand the real-life experiences and needs of older adult patients with Parkinson’s disease and their caregivers regarding home-based exercise rehabilitation, and to provide evidence for developing scientifically grounded home exercise rehabilitation intervention programs.

**Methods:**

A total of 15 older adult patients with Parkinson’s disease and 13 caregivers from the Neurology Department of a tertiary-level Class A hospital in Urumqi were selected using purposive sampling for semi-structured interviews. Data analysis and theme extraction were conducted using content analysis.

**Results:**

The analysis identified three primary themes: (1) “The Knowledge–Practice Gap in Self-Management,” in which poor understanding of PD and exercise benefits resulted in low motivation and a lack of accessible skills led to monotonous routines; (2) “The Unsupported Caregiver: A System and Family Gap,” in which the efforts of caregivers were hindered by their own limitations and a lack of training, compounded by the absence of family and community support networks; and (3) “An Appeal for an Integrated Support Continuum,” which reflected a strong need for ongoing professional guidance and comprehensive social and policy support.

**Conclusion:**

Systemic multidimensional bottlenecks, specifically a critical knowledge–practice gap among patients and a stark lack of support for aging caregivers, hinder home-based exercise rehabilitation for older adults with PD in northwest China. To address these context-specific barriers, healthcare systems should develop an integrated “hospital–community–family” care model that provides continuous professional guidance and robust social support, ultimately aiming at improving rehabilitation efficacy and quality of life.

## 1 Introduction

Parkinson’s disease (PD) is a common neurodegenerative disorder (1). Recently, the annual prevalence of PD has increased. With the acceleration in population aging, the global number of patients with PD is projected to increase to 10 million by 2030 (2). The primary clinical manifestations of PD include tremors, muscle rigidity, bradykinesia, postural instability, dysarthria, dysphagia, and sleep disorders (3). Medication is currently the primary treatment option for patients with PD. However, long-term use is often accompanied by adverse reactions, and treatment efficacy becomes limited as the disease progresses (4). Research indicates the potential of exercise therapy in improving multiple clinical symptoms of PD (5), and it is often combined with medication in clinical practice to enhance its efficacy. In China, particularly in regions such as Xinjiang, home-based rehabilitation is an important long-term management modality for several older adults with PD who have limited access to continuous professional rehabilitation services. As suggested by national expert consensus, standard home-based exercise protocols often encompass gait and balance training, flexibility exercises, muscle strengthening, and “activity of daily living” training (6). Nonetheless, the translation of these guidelines into practice faces significant challenges, including a lack of personalized adaptation to patients’ physiological conditions and home environments, insufficient community-based support systems, and limited resources for tailored supervision (7). This results in low patient adherence and suboptimal rehabilitation outcomes. For older adult patients in particular, home-based exercise rehabilitation is a crucial modality that relies heavily on caregiver support and cooperation. However, existing research has not systematically explored caregivers’ actual experiences and specific needs during the assistance process, even though the quality of caregiver support directly affects the effectiveness of home-based exercise rehabilitation. Therefore, this study aims to use semi-structured interviews to gain an in-depth understanding of the experiences and needs of older adult patients with PD and their caregivers during home-based exercise rehabilitation. This study aimed to provide a basis for developing scientific, personalized, and home-based exercise rehabilitation intervention plans.

## 2 Methods

### 2.1 Participants

Using purposive sampling, 15 older adult patients with PD and 13 primary caregivers from the neurology department of a tertiary hospital in Urumqi were selected for semi-structured interviews. The sample size was determined based on the principle of data saturation in qualitative research, aiming to achieve a comprehensive understanding of the research topic rather than statistical representativeness. Recruitment continued until no new themes or insights emerged from consecutive interviews, which was achieved after interviewing 15 patients and 13 caregivers. Patient inclusion criteria: (1) Age ≥60 years, meeting the 2016 Chinese diagnostic criteria for PD (8); (2) Hoehn & Yahr stage I–III; (3) Clear consciousness, basic communication ability, and voluntary participation. The exclusion criteria were as follows: (1) Parkinson’s superimposed syndrome or secondary Parkinsonism; (2) Severe cardiopulmonary disease, stroke, malignant tumors, or other contraindications. Caregiver inclusion criteria: (1) Continuous caregiving duration ≥6 months; (2) Informed consent and voluntary participation in this study. The exclusion criteria were as follows: (1) Hearing impairment or speech communication difficulties; (2) Personal history of severe chronic diseases (e.g., malignant tumors); (3) Inability to cooperate due to other reasons. This study enrolled 15 older adult patients with PD and 13 caregivers. The mean age of patients was (71.07 ± 7.20) years, coded as N1–N15. The mean age of caregivers was (57.85 ± 7.60) years, coded as P1–P13. All participants signed informed consent forms. This study received hospital ethics approval: KY2025062408. It is important to note that although the findings provide in-depth insights, purposive sampling from a single center may limit the generalizability of findings to the broader PD population in different cultural or healthcare contexts. Demographic details of the participants are presented in Tables 1, 2.

**Table 1:** General patient information (*N* = 15).

NO.	Age	Gender	Educational attainment	Marital status	Combined chronic diseases	Number of falls in the past year	Phases
N1	66	Male	Junior high school	Married	Diabetes	0	1.5
N2	80	Male	High school	Married	Hypertension, diabetes, coronary heart disease	0	2
N3	68	Male	Junior high school	Married	None	0	1
N4	60	Male	vocational high school	Married	None	0	1
N5	60	Female	High school	Married	Diabetes	0	1.5
N6	71	Female	Junior high school	Married	None	0	3
N7	87	Male	Junior high school	Married	Chronic obstructive pulmonary disease	1	3
N8	71	Male	Junior High School	Married	Hypertension, chronic kidney disease	0	2
N9	69	Female	High school	Widowed	None	0	2
N10	80	Female	Junior high school	Married	None	0	1.5
N11	68	Female	Junior high school	Married	Coronary heart disease	0	1.5
N12	68	Female	Elementary school	Married	None	0	3
N13	72	Male	High school	Married	None	0	1.5
N14	73	Male	Junior high school	Married	Hypertension	0	2.5
N15	73	Female	Elementary school	Married	Diabetes	2	2.5

**Table 2:** General characteristics of caregivers (*N* = 13).

NO.	Age	Gender	Educational attainment	Patient relationship	Care time (years)	Physical condition
P1	57	Female	Junior high school	Daughter-in-law	2	Fair
P2	53	Female	Junior high school	Daughter	1	Good
P3	61	Female	High school	Spouse	2	Fair
P4	66	Female	Junior high school	Spouse	1	Fair
P5	55	Female	High school	Spouse	5	Good
P6	54	Female	Junior college	Daughter	3	Fair
P7	50	Male	High school	Son	1	Good
P8	51	Female	High school	Spouse	2	Good
P9	73	Male	Junior High School	Spouse	3	Fair
P10	51	Female	Junior college	Daughter	3	Good
P11	70	Female	Junior high school	Spouse	2	Fair
P12	52	Female	High school	Daughter	2	Good
P13	59	Male	Junior college	Spouse	2	Fair

### 2.2 Data collection methods

This study employed a phenomenological qualitative research approach. All interviews were carried out by the primary researcher, who is a master’s prepared nurse with training in using qualitative methods and no prior clinical relationship with the participants. Based on a systematic review of relevant literature and research objectives, the research team developed a preliminary interview outline through group discussions. To validate the applicability of the outline and refine the questions, the study first conducted pre-interviews with two patients and two caregivers who met the inclusion and exclusion criteria. The final formal interview guide was developed based on feedback from preliminary interviews. The patient interview guide was as follows: (1) Could you describe your daily activities in terms of physical activity and exercise? (2) Please discuss the role you believe exercise plays in home rehabilitation. (3) What is the inner experience like for you when you engage in your home exercises? (4) Could you describe a specific time when you found home-based exercise particularly challenging or particularly rewarding? (5) When considering continuing home exercise, what aspects do you find difficult? (6)What kind of support or resources would make your home exercise journey feel more manageable or meaningful?(7) Besides healthcare providers, what support would you like from family, community, or other sources? The caregiver interview outline was as follows: (1) Can you walk me through your typical day supporting your family member?(2) Describe the experience of assisting with exercises from your perspective. What moments stand out to you?(3) What has been the most significant challenge for you in this role, and how has it impacted you? (4) From a broader societal perspective (e.g., community services, policy support), what support would alleviate your caregiving burden? This study employed semi-structured one-on-one interviews. Prior to each interview, participants were informed of the study’s purpose and main topics, with assurances of confidentiality for all content. Interviews proceeded only after informed consent was obtained. In each session, the researchers utilized active listening and probing techniques (e.g., “Can you tell me more about that?”) to facilitate in-depth dialogue and also avoided leading questions while simultaneously documenting both verbal and nonverbal cues. All interviews were conducted in a quiet ward within the hospital. Each session lasted for 20–30 min and was fully audio-recorded.

### 2.3 Data analysis methods

Qualitative data were analyzed using content analysis (9). (1) Interview transcripts were produced within 24 h of each interview and carefully reviewed multiple times. (2) During the review process, meaningful statements relevant to the research questions were extracted. (3) The extracted statements were inductively compared and open-coded; subsequently, the codes were pooled to identify recurring viewpoints and form preliminary themes. (4) The preliminary themes underwent review, comparison, and refinement. Viewpoints with similar expressions or inherent consistency were consolidated and categorized into distinct thematic groups based on their characteristics, establishing a clear thematic structure. (5) Link the finalized themes to the original narratives of research subjects, providing detailed descriptions and interpretations to ensure analytical findings authentically and accurately reflect interviewees’ original intentions. (6) Through systematic analysis of all themes and their integration with research questions, derive the analytical conclusions of this study.

## 3 Results

### 3.1 The knowledge-practice gap in self-management

#### 3.1.1 Poor understanding of PD and exercise benefits results in low motivation

Most older adult patients with PD and their caregivers reported that their understanding of PD was generally acquired after diagnosis. They were aware only of medication treatment and were unfamiliar with exercise rehabilitation methods; thus failing to systematically engage in home-based exercise rehabilitation. N3: “My daughter noticed my head would twitch involuntarily sometimes and urged me to get checked. That’s how I learned about Parkinson’s disease. All my knowledge about it came from the doctor, and I knew nothing about exercise rehabilitation.” N7: “We never imagined it could be Parkinson’s disease. We just thought it was a normal part of aging. We did not understand it and never did any exercise rehabilitation.” P2: “We only learned about this disease after our family member became ill. We only know about medication treatment and are unaware of any targeted home-based exercise rehabilitation methods. We have not done any exercise rehabilitation either.”

#### 3.1.2 Lack of accessible knowledge and skills leads to simplistic and unsustainable routines

Even when exercising, the vast majority of patients confine their rehabilitation activities to daily walks, resulting in a monotonous routine. Respondents reported feeling that such exercise “does not make much of a difference” or “does not seem to improve their physical condition.” However, lacking awareness of other targeted rehabilitation methods, they are unable to alter this situation, significantly diminishing the effectiveness of their recovery. N2: “Usually I walk for half an hour at home, but walking every day does not make me feel anything. I used to paint at home, but now my hands shake and I can no longer paint anymore.” N5: “My usual exercise is walking for 30 min with my spouse, but doing this every day does not make me feel any improvement in my body. I do not know about other rehabilitation exercises either. I just look online to see if there are any new drugs that can cure it.” N9: “I do not exercise or work out. I sometimes walk at home, but not every day.”

### 3.2 The unsupported caregiver: a system and family gap

#### 3.2.1 Caregivers’ efforts are hindered by their own limitations and a lack of training

This study revealed that spouses or children assuming caregiving responsibilities face challenges such as advanced age, poor health, and lack of knowledge, resulting in limited caregiving capacity. They admit that daily care is already a struggle, and when it comes to movement rehabilitation assistance requiring specialized expertise, they feel “willing but unable.” P3: “We’re getting older too, and our children all have jobs. We can only manage daily living—anything beyond that is beyond our means.” P7: “We children are also getting older and have our own families. We can only take turns providing basic care. When it comes to assisting with rehabilitation, we are willing but unable—we simply do not understand it ourselves”.

#### 3.2.2 Absence of robust family and community support networks exacerbates the caregiving burden

Inadequate family support manifests in two aspects. First, family members lack knowledge of rehabilitation, preventing them from providing effective assistance and supervision. Second, limited financial resources and physical environments (such as the absence of safety handrails and rehabilitation equipment) hinder safe and effective home-based rehabilitation training, creating a practical dilemma of “inability to implement.” N9: “Exercising at home is not safe either—we do not have specialized handrails or rehabilitation equipment.” P10: “Our family also faces heavy financial burdens. We cannot afford specialized home modifications or supervision to assist with his exercise rehabilitation, nor can we hire professionals for rehabilitation services.”

### 3.3 An appeal for an integrated support continuum

#### 3.3.1 A cry for professional, ongoing, and accessible rehabilitation guidance

Most patients and caregivers expressed a strong desire for knowledge about home-based exercise rehabilitation. While they are willing to exercise, they struggle with “not knowing the methods.” They urgently hope professionals can “teach them how to exercise” and seek diverse professional support ranging from community rehabilitation guidance to in-home services. N6: “I feel that if someone just teaches me how to do it and what precautions to take, I can perform the exercises.” N13: “We do not know what exercise methods are available. We can walk now, and we’d be willing to exercise if we learned how—it’s just that we do not know.” P5: “We’ve bought books to learn on our own, but we lack professional guidance. Could the community provide some rehabilitation equipment and introduce professional rehabilitation therapists?”

#### 3.3.2 A call for comprehensive social and policy support to share the burden of care

To alleviate caregiving burdens, respondents expressed a strong desire for broader social support. This includes hopes for centralized rehabilitation services and in-home guidance from communities or professional institutions. They also seek policy-based support, such as long-term care insurance, to substantially ease the financial and physical strains that families endure during caregiving. N3: “We do not know how to conduct exercise rehabilitation. Could specialized exercise rehabilitation centers be established where we could go daily for workouts or schedule home visits with professionals for exercise guidance?” N8: “We hope the community can provide weekly or concentrated home visits to older adult patients with this condition, offering professional exercise rehabilitation and care services.” P12: “The burden of caring for older adult patients is growing increasingly heavy. We hope for measures like long-term care insurance, longevity insurance, and home-based exercise rehabilitation to alleviate the caregiving burden.”

## 4 Discussion

Research findings indicate that although some patients possess a certain level of awareness about PD itself after diagnosis, their understanding of home-based exercise rehabilitation remains limited. This results in monotonous rehabilitation activities and insufficient participation, thereby affecting overall rehabilitation outcomes. This aligns with studies by Xie (10). The root causes lie partly in the systematic lack of knowledge about disease management among patients and their families, reflecting a global phenomenon in which patients often feel ill-prepared for self-management after discharge (11). This suggests a structural bias in current rehabilitation health education that prioritizes hospital settings over home environments. Furthermore, our data highlight a specific structural bias within the Chinese healthcare system—a model that prioritizes acute hospital-based interventions over chronic community-based management. Standardized exercise therapy has been shown by previous studies to effectively improve motor symptoms and delay functional decline in patients with PD, thereby enhancing their quality of life (12–14). Nevertheless, our context reveals a reliance on standardized, one-size-fits-all handouts that lack personalization. Therefore, strengthening inpatient education is necessary but insufficient. Healthcare providers must leverage this critical window to deliver structured hands-on training and establish peer support groups, which are effective in enhancing self-efficacy and adherence in international practice (15). Healthcare providers should also strengthen the systematic education on exercise rehabilitation during the inpatient phase. This should include the performance of typical case studies to improve patient understanding, as well as the provision of hands-on instruction to help patients master basic rehabilitation skills. Additionally, establishing peer support groups can leverage peer education and foster a rehabilitation environment of mutual supervision and motivation, thereby improving adherence to and continuity of home-based rehabilitation.

A central finding of this study is the precarious position of caregivers, who commonly feel “willing but unable” to assist due to their own advanced age, health issues, and knowledge deficits. This corroborates the work of Mao (16). and finds a powerful echo in the international literature on caregiver burden, which consistently highlights the correlation between the caregiver’s own health, their perceived burden, and patient outcomes (17). The situation in China, however, is exacerbated by its rapidly ageing population and the legacy of the one-child policy, which has shrunk the pool of available family caregivers. The “4-2-1” family structure places an immense responsibility on a single individual. Our discussion of “weak family support systems” must be framed within this broader demographic and policy context. The current lack of comprehensive long-term care insurance (LTCI) and subsidized respite care services means that the burden falls almost entirely on the family, creating a silent public health crisis (18, 19). Therefore, recommendations must extend beyond skill training. Community health centers should integrate geriatric care principles into their support programs, Furthermore, the recommendation for public exercise facilities is intended to indirectly alleviate caregiver burden. By providing a safe, community-based venue, these facilities can shift the context of exercise supervision from the home to the community, offering caregivers temporary respite and reducing the physical demand of assisting with exercises at home. Policymakers must accelerate the piloting and expansion of LTCI to provide financial and service support, thereby alleviating the foundational pressures on caregivers.

This study also revealed the multifaceted demands of patients and caregivers for specialized, continuous, and accessible rehabilitation support. These include remote guidance, community rehabilitation resources, home-visit services, and policy safeguards, reflecting the current gaps in service continuity between hospitals, communities, and homes. It is crucial to clarify that these are not competing but complementary strategies. Home-based interventions are the foundational element for daily practice, while community-based services and resources provide the necessary supervision, social engagement, and professional support that make sustained home-based care feasible and effective (20). This study provides qualitative evidence for the urgent need to operationalize the “hospital-community-family” integrated care model that is a stated goal of China’s healthcare reform (21). The proposed solutions, such as developing a PD rehabilitation app, establishing community clinics, and creating “rehabilitation points” incentive mechanisms, are concrete steps toward building this continuum. Specifically, the role of tertiary hospitals must evolve from being the sole provider to being the leader of a network, responsible for training community physicians and establishing standardized remote guidance protocols (22). Community health centers should be empowered and funded to function as the operational hub, coordinating home-visit services and managing group rehabilitation sessions. Expanding LTCI (23) is not merely a financial tool but a critical enabler for this integrated model, as it can formally reimburse community-based rehabilitation services that are currently scarce or out-of-pocket expenses.

## 5 Conclusion

Through an in-depth qualitative exploration, this study moves beyond generic challenges to reveal the fundamental disconnect between knowledge and practice that characterizes home-based exercise rehabilitation for PD patients in Northwest China. It uniquely highlights the precarious position of the ageing, unsupported caregiver as a critical barrier, a finding acutely shaped by local demographic pressures. Rather than merely listing needs, our findings provide a qualitative evidence base for the urgent development of an integrated hospital-community-home care model, offering concrete, participant-driven insights—such as the call for professional guidance and social policy support—to inform the creation of tailored intervention protocols. To validate these context-specific findings, future research should expand to multi-center samples and employ mixed-methods approaches.

## Data Availability

The original contributions presented in the study are included in the article/supplementary material, further inquiries can be directed to the corresponding author.
